# Autofluorescence findings and role of anti-vascular endothelial growth factor in inflammatory choroidal neovascular membrane

**DOI:** 10.4103/0301-4738.67041

**Published:** 2010

**Authors:** Jay Kumar Chhablani

**Affiliations:** L V Prasad Eye Institute, Hyderabad, India

Dear Editor,

I read the article by Dhingra *et al*.,[1] which describes the pathogenesis and treatment of the inflammatory choroidal neovascular membrane (CNVM). I would like to add a few comments about the autofluorescence (AF) findings and use of the anti-vascular endothelial growth factor (VEGF) in inflammatory CNVM.

Autofluorescence in Inflammatory CNVM

Many reports showed the utility of AF as a marker for early disease and in predicting the outcome of treatment in CNVM due to age-related macular degeneration. There are very few reports on AF findings in inflammatory CNVM.

Inflammatory CNVM, which is usually of the classic type (type 2), is seen on AF photography as a precise hyper-autofluorescent area due to the hyperplastic retinal pigment epithelium (RPE). Following treatment, CNVM may contract and leave a zone of absent RPE causing a hypo-autofluorescence.

Secondary choroidal neovascularization in multifocal choroiditis and panuveitis (MCP) is readily visible as a hyper-autofluorescencent area originating from a hypo-autofluorescent spot (scar). With increasing follow-up time, the hyper-autofluorescence associated with CNV decreases. AF imaging provides a method to image the appearance of new or enlarging spots that appear, which is more sensitive than using ophthalmoscopically visible signs.

More studies are required to understand the role of AF in inflammatory diseases. AF imaging could be an important noninvasive tool to reduce the need for angiography and to help in early diagnosis, as also in the follow-up of inflammatory CNVM patients.

Use of Anti Vegf0 in Inflammatory CNVM

There are potential disadvantages of treating inflammatory CNV with photodynamic therapy (PDT). PDT can cause localized inflammation and increase VEGF production. Local release of VEGF, after PDT, may be associated with a higher incidence of recurrent choroidal neovascularization (CNV).[2]

Tran *et al,* studied 10 patients with CNV, who were refractory to previous immunosuppression and PDT or intravitreal triamcinolone (IVTA). They reported that intravitreal bevacizumab improved the best corrected visual acuity (BCVA) and reduced the central macular thickness in the eyes with 2.5 injections (mean), and this was seen at 7.5 months of follow up. In this study they included a patient who recurred three months after pegaptanib injection and responded well.[3] Fine *et al* reported successful treatment of inflammatory CNVM with ranibizumab in a patient with multifocal choroiditis and pan uveitis (MCP), who did not responded to bevacizumab or the PDT and IVTA therapy.[4]

The recent and largest case series by Monsour *et al* reported significant improvement of 2.2 lines in BCVA at 24 months, with a significant decrease in foveal thickness (265 microns), with only 1.3 injections (mean). This study confers the long-term benefits of intravitreal bevacizumab.[5]

Serious side effects and chances of recurrence make PDT an obsolete treatment option. Recent promising results favor intravitreal bevacizumab as a primary / mono therapy in inflammatory CNVM.
